# Novel Green Synthesis Route of ZnO Nanoparticles for Dielectric Applications

**DOI:** 10.3390/nano15130991

**Published:** 2025-06-26

**Authors:** Zohra Benzarti, Joana Neiva, Pedro Faia, Eduardo Silva, Sandra Carvalho, Susana Devesa

**Affiliations:** 1 Centre for Mechanical Engineering, Materials and Processes (CEMMPRE)-Advanced Production and Intelligent Systems, Associated Laboratory (ARISE), Department of Mechanical Engineering, University of Coimbra, Rua Luís Reis Santos, 3030-788 Coimbra, Portugal; uc2023148579@student.uc.pt (J.N.); sandra.carvalho@dem.uc.pt (S.C.); 2 Laboratory of Multifunctional Materials and Applications (LaMMA), Department of Physics, Faculty of Sciences of Sfax, University of Sfax, Soukra Road km 3.5, Sfax 3000, Tunisia; 3 Centre for Mechanical Engineering, Materials and Processes (CEMMPRE), Advanced Production and Intelligent Systems (ARISE), Electrical and Computer Engineering Department, FCTUC, University of Coimbra, Pinhal de Marrocos, 3030-290 Coimbra, Portugal; faia@deec.uc.pt; 4 Durit Coatings, Parque Industrial de Taveiro nº 41 e 42, 3045-508 Coimbra, Portugal; innovation@duritcoatings.pt; 5 Laboratory of Tests, Wear and Materials, IPN-LED & MAT-Instituto Pedro Nunes, Rua Pedro Nunes, 3030-199 Coimbra, Portugal

**Keywords:** ZnO nanoparticles, green synthesis, *Hylocereus undatus*, dielectric properties

## Abstract

This study presents a novel, eco-friendly synthesis route for zinc oxide (ZnO) nanoparticles using cladode extracts of *Hylocereus undatus* acting simultaneously as reducing and improving agents, in alignment with green chemistry principles. The synthesis involved the reaction of zinc sulfate heptahydrate with the plant extract, with the medium pH adjusted using sodium hydroxide (NaOH), followed by calcination at 300 °C, 400 °C, and 500 °C, and then by a washing step to enhance purity. Comprehensive characterization was performed using thermogravimetric analysis (TGA), differential scanning calorimetry (DSC), X-ray diffraction (XRD), Raman spectroscopy, scanning electron microscopy (SEM), energy-dispersive X-ray spectroscopy (EDS), and electrical impedance spectroscopy to investigate the structural, morphological, and dielectric properties of the nanoparticles. The sample calcined at 400 °C, followed by washing (HT400W), exhibits highly crystalline ZnO nanoparticles with a predominant wurtzite structure (93.15 wt% ZnO) and minimal impurities (6.85 wt% Na_2_SO_4_). SEM analysis indicated a flake-like morphology with nanoscale features (50–100 nm), while Raman spectroscopy confirmed enhanced crystallinity and purity post-washing. Additionally, the HT400W sample exhibited a dielectric constant (ε′) of 16.96 and a low loss tangent (tan δ) of 0.14 at 1 MHz, suggesting superior energy efficiency for high-frequency applications. This green synthesis approach not only eliminates hazardous reagents but also delivers ZnO nanoparticles with good dielectric performance. Furthermore, this work demonstrates the efficacy of a sustainable biotemplate, offering an environmentally friendly approach for synthesizing ZnO nanoparticles with tailored physicochemical properties.

## 1. Introduction

The movement towards eco-friendly practices has driven notable progress in nanotechnology [1]. In conventional nanoparticle synthesis, two primary methods are commonly employed: top-down and bottom-up. The top-down method involves breaking down bulk materials into nanoparticles using techniques such as lithography, ball milling, etching, and sputtering. On the other hand, the bottom-up approach builds nanoparticles from atoms or molecules through processes like chemical vapor deposition, sol-gel-based methods, spray pyrolysis, laser pyrolysis, and atomic or molecular condensation [2,3,4].

Conventional techniques for synthesizing nanoparticles, though effective, frequently involve toxic substances, substantial energy requirements, and intricate procedures that can threaten human health and the environment [5,6]. In comparison, biosynthesis provides an environmentally friendly option, utilizing biological substances like plants, algae, bacteria, and fungi to create nanoparticles [7,8,9]. Among these, the synthesis mediated by plants has attracted significant interest because of its ease of access, simplicity, scalability, and dual role of plant extracts as reducing and stabilizing agents in the process of nanoparticle (NPs) formation [10,11].

Phytochemicals such as flavones, organic acids, ketones, aldehydes, and amides present in plant extracts effectively convert metal ions into nanoscale forms. Compounds that dissolve in water, such as flavones, organic acids, and quinones, facilitate the reduction of zinc ions, while benzoquinones like cyperoquinone, dietchequinone, and remirin assist in reducing particle size. This combination of reducing and stabilizing functions makes plant extracts a reliable and eco-friendly choice for the synthesis of green NPs [12,13]. In recent years, various kinds of plant extracts have been documented as reducing or capping agents in the production of NPs. Several examples encompass the environmentally friendly production of zinc oxide (ZnO) NPs through waste peel extract [14], moringa seeds [1], and sea lavender [13].

Dragon fruit (*Hylocereus* spp.), also known as Pitaya or Pitahaya from the Cactaceae family [15], originally from the tropical regions of Central Mexico, and South America [16], has gained focus due to the high economic value of its fruit [17].

Although 14 species of *Hylocereus* have been identified worldwide, the predominantly cultivated species are *H. undatus* (pink skin/white flesh), *H. monocanthus* (*Syn. H. polyrhizus*) (red flesh/pink skin), *H. costaricencis* (violet-red flesh/pink skin), *H. guatemalensis*, (red flesh/reddish-orange skin), and *H. megalanthus* (*Syn. H. selenicerus*) (white flesh/yellow skin) [18,19,20].

While earlier research has investigated *Hylocereus undatus* to produce zinc oxide nanoparticles, a significant difference in the mentioned study is the use of fruit peel extracts [21], in contrast to the use of cladodes in this research. It is important to note that cladodes have demonstrated superior performance, as indicated by Tsai et al. [22], who assessed the phenolic compound levels in different parts of *Hylocereus polyrhizus* and consistently observed that cladode extracts were more effective than those from flowers or fruits. This emphasizes the capability of cladodes to produce phytopreparations from dragon fruit byproducts.

ZnO nanoparticles have emerged as a significant focus of research due to their unique physicochemical and dielectric properties, featuring a large band gap (3.37 eV), dimensions between 1–100 nm, and substantial exciton binding energy (60 meV), allowing the material to function at considerably higher voltage, frequency, and temperature than the observed for traditional semiconductors [14,23,24]. These nanoparticles exhibit superb thermal conductivity, impressive refractive indices, notable binding energies, and exceptional UV-blocking capabilities.

These characteristics have established their importance in numerous applications, including energy storage, dielectric spectroscopy, photocatalysis, chemical sensing, solar energy, and biomedicine [25,26,27,28,29]. An emerging area of study is the one focused on examining the dielectric properties of ZnO, which are crucial for energy storage systems and electronic devices. By enhancing these attributes, ZnO-based materials can significantly boost the performance and efficiency of capacitors, batteries, and other energy storage systems [23,30,31].

In this research, cladode extracts of *Hylocereus undatus* were employed as a novel eco-friendly template for the synthesis of ZnO nanoparticles. *Hylocereus undatus* is a cactus species prized for its health and dietary benefits, rich in natural phytochemicals that act as potent reducing and capping agents. These extracts not only streamline the synthesis procedure but also eliminate the need for hazardous reagents, adhering to the principles of green chemistry. The synthesized ZnO nanoparticles underwent comprehensive characterization to assess their dielectric properties, highlighting their potential applications in energy storage. This study seeks to connect sustainable material manufacturing with cutting-edge technological applications by emphasizing the creative application of *Hylocereus undatus* cladodes extracts in the producing of nanoparticles. Moreover, it underscores the broader significance of employing environmentally conscious synthesis techniques in creating high-performance materials across various domains, including electronics and biomedicine.

## 2. Materials and Methods

### 2.1. Materials

The cladodes of *Hylocereus undatus* were harvested from a mature plant in a garden located in Coimbra, Portugal, during October.

For the synthesis of ZnO nanoparticles, in addition to the *Hylocereus undatus* cladode extract, zinc sulfate heptahydrate (ZnSO_4_·7H_2_O, ≥99.5%, Labkem, Baldoyle, Ireland) and sodium hydroxide (NaOH, 98%, Panreac, Barcelona, Spain) were used. The deionized water employed was produced in-house using ultra-pure water equipment.

### 2.2. Preparation of the Hylocereus undatus Cladodes Extract

After spine removal, the *Hylocereus undatus* cladodes were thoroughly washed, first with tap water, followed by deionized water, and cut into small pieces. A mixture of 250.0 mL of deionized water and 60.7 g of cladode pieces was heated and kept at 80 °C for 2 h. After an 18-h resting period, the mixture was filtered through a 45 μm sieve and stored for future use.

### 2.3. Synthesis of the ZnO Nanoparticles

The plant extract was added dropwise to 25.00 g of ZnSO_4_·7H_2_O, promoting its complete dissolution. This procedure was performed at room temperature under magnetic stirring at 200 rpm. While maintaining the stirring conditions, an aqueous solution of NaOH (0.1 wt%) was dropwise added, allowing the increment of the pH level of the previous solution from 3.74 to 11.00. The mixture was then heated to 80 °C and stirred until the solvent evaporated, a process that took 4 h.

**Figure 1:**
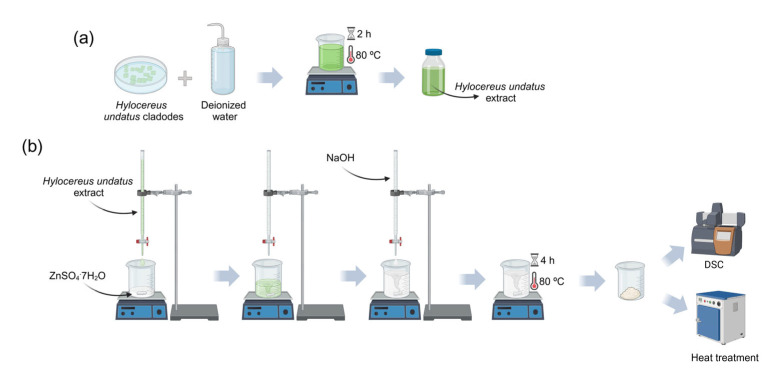
Schematic representation: (**a**) preparation of *Hylocereus undatus* cladode extract; (**b**) synthesis of ZnO nanoparticles (Created in BioRender. Devesa, S. (2025) https://BioRender.com/c92u638, accessed on 21 June 2025).

For calcination, three different temperatures were selected based on the thermal characterization performed: 300 °C, 400 °C, and 500 °C. The heat treatments were conducted in a furnace under an air atmosphere for 2 h, using for all cases a heating rate of 5 °C/min. Figure 1 illustrates the flowchart of the steps involved in the described synthesis.

According to the structural characterization, specifically the phase composition determined for each sample, the most promising one was subjected to a washing process with deionized water in an ultrasonic bath to remove soluble byproducts formed during the synthesis process. Afterwards, the resulting powder was heated and maintained at 150 °C for 3.5 h to remove the deionized water.

### 2.4. Characterization Techniques

Thermogravimetric analysis (TGA) was performed on a Netzsch TG 209 F1 Libra (Selb, Germany) in a nitrogen atmosphere, with a heating rate of 5 °C/min, ranging from 30 to 1095 °C. Differential scanning calorimetry (DSC) was carried out between 25 and 500 °C using a Netzsch DSC 204 F1 Phoenix (Selb, Germany), also in a nitrogen atmosphere, utilizing the same heating rate of 5 °C/min.

X-ray diffraction (XRD) analysis was performed using a Rigaku Smartlab diffractometer (Tokyo, Japan) with Cu Kα (λ = 1.54060 Å) radiation, operating at 40 kV and 50 mA, utilizing a Bragg Brentano geometry, with a step size of 0.02°. To confirm the crystal structure and acquire supplementary structural information about the samples, Rietveld refinement was carried out using Profex [32].

Raman spectroscopy measurements were executed with a Renishaw inVia™ (Wotton-under-Edge, UK) confocal Raman spectrometer, employing a 532 nm green laser.

The morphology of the sintered powders was examined by scanning electron microscopy (SEM), using a Zeiss Merlin microscope (Zeiss, Oberkochen, Germany), in secondary electron mode.

The elemental composition was analyzed via energy-dispersive X-ray spectroscopy (EDS) using a Bruker Nano System (Berlin, Germany), set at an accelerating voltage of 15 kV.

Finally, electrical impedance spectroscopy measurements were performed using an Agilent 4294A (Agilent Technologies, Santa Clara, California, USA) in the C_p_-R_p_ configuration, in air and at room temperature, over a frequency range between 50 Hz to 10 MHz.

To perform sample evaluation (for which two acquisition runs were conducted), the powders were uniaxially pressed to obtain pellets, and silver electrodes were applied to the surfaces of the pellets using silver ink from SPI Supplies (West Chester, PA, USA). Measured complex impedance data integrity was ensured by the Agilent equipment, which includes inbuilt Kramer–Krönig transformations [33].

## 3. Results

### 3.1. Characterization of Hylocereus undatus Cladodes

**Figure 2:**
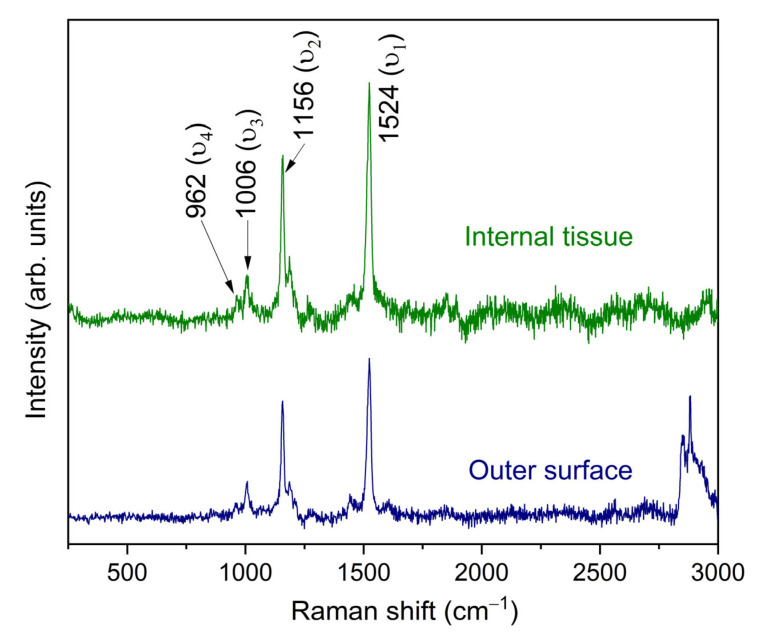
Raman spectra of the outer surface and internal tissue of the cladodes of the *Hylocereus undatus*.

The Raman spectra of both the outer surface and internal tissue of the *Hylocereus undatus* cladodes, presented in Figure 2, reveal distinctive vibrational modes indicative of their biochemical composition, particularly carotenoids and structural carbohydrates. In concrete, the spectrum for the internal tissue shows three prominent bands centered at approximately 1006 cm^−1^, 1156 cm^−1^, and 1524 cm^−1^, which are characteristic of carotenoids, consistent with findings for Opuntia ficus-indica cladodes [34]. The band at 1006 cm^−1^ ($\vartheta_{3}$) corresponds to C–CH_3_ in-plane rocking deformations coupled to C–C bonds, reflecting methyl side chains in carotenoids [35]. The band at 1156 cm^−1^ ($\vartheta_{2}$) arises from a combination of C–H in-plane bending and C–C stretching vibrations along the polyene chain, while the band at 1524 cm^−1^ ($\vartheta_{1}$) is attributed to the stretching vibrations of the conjugated C=C backbone of carotenoids, a hallmark of their polyene structure. These assignments align with the Raman signatures of carotenoids observed in plant tissues, as reported for cacti and other species [35,36]. Below 1000 cm^−1^, a weaker band at approximately 962 cm^−1^ ($\vartheta_{4}$) is observed in the internal tissue spectrum, likely arising from out-of-plane motions of hydrogen nuclei on the conjugated chain, which can intensify with steric hindrance or structural distortions, such as those within protein-binding pockets [35].

In contrast, the Raman spectrum of the outer surface shows broader and less intense peaks, with the same dominant bands at ~1006 cm^−1^, ~1156 cm^−1^, and ~1524 cm^−1^, but with reduced clarity and intensity. This difference suggests a lower concentration or altered arrangement of carotenoids in the outer surface, possibly due to environmental exposure (e.g., UV radiation, desiccation) or the presence of a protective cuticle, which may mask or modify the Raman signal. Additionally, the outer surface may contain higher levels of waxy or lignin-like compounds [37]; indeed, the broad band between 2820 cm^−1^ and 3000 cm^−1^ observed exclusively in the Raman spectrum of the outer surface of *Hylocereus undatus* cladodes is attributed to symmetric C–H stretching vibrations, characteristic of aliphatic hydrocarbons in lipids or waxes present in the protective cuticle. This cuticle, rich in long-chain hydrocarbons and waxes, is a key feature of the outer surface, protecting against environmental stressors like desiccation and UV radiation, and is absent or minimal in the internal tissue [37].

### 3.2. Characterization of ZnO Nanoparticles

**Figure 3:**
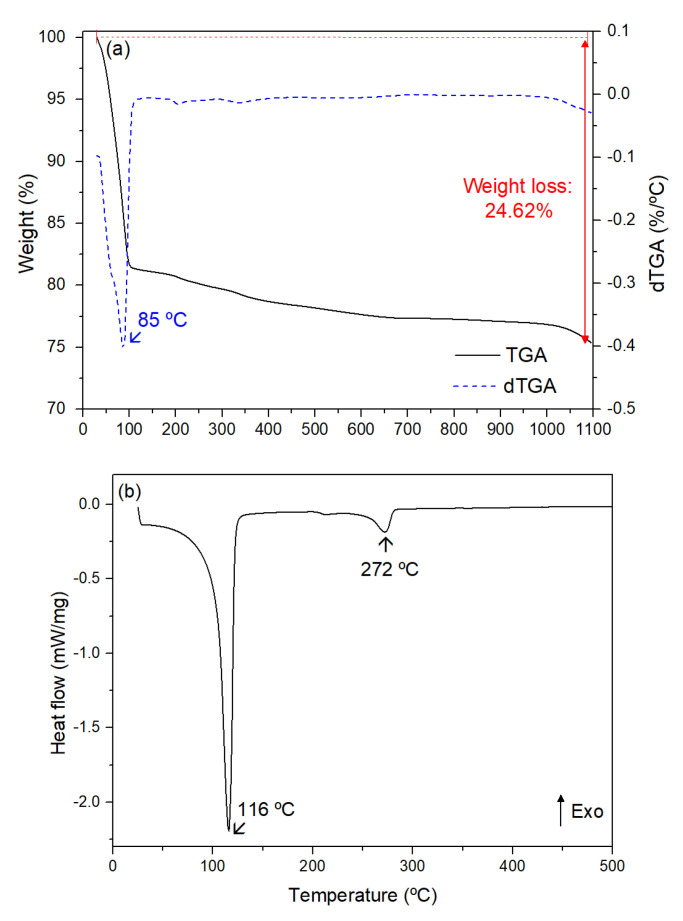
(**a**) TGA, dTGA, and (**b**) DSC curves of the synthesized powder.

Figure 3a shows the TGA and derivative TGA (dTGA) curves of the biosynthesized powder, where a total weight loss of 24.62%, mainly occurring up to around 85 °C, can be seen.

The DSC curve, depicted in Figure 3b, exhibits two endothermic peaks centered at about 116 and 272 °C.

The first peak is assigned to the vaporization of the physically absorbed water [38,39,40], while the endothermic peak that appears at 272 °C is likely due to the formation of ZnO nanoparticles and to the degradation of organic matter [39,40,41]. These inferences are in accordance with the TGA results, once the vaporization of the physically absorbed water can be associated with a significant mass loss, while the formation of a new crystalline phase is linked to a minor mass loss.

Based on this thermal analysis and to understand the effect of calcination temperature on the ZnO nanoparticles, three heat treatment temperatures were selected: 300 °C, 400 °C, and 500 °C.

A heat treatment at 300 °C ensures the removal of physically adsorbed species and thermally unstable components, while the heat treatments at 400 °C and 500 °C are expected to promote further structural stabilization and the gradual enhancement of crystallinity, given the thermal stability observed beyond 300 °C and the absence of significant decomposition or thermal transitions in the TGA and DSC curves.

Hereafter, the samples will be referred to as HT, followed by the corresponding treatment temperature, and W, indicating the washing process if applicable (e.g., HT400 for the sample treated at 400 °C and HT400W for the sample washed after heat treatment).

**Figure 4:**
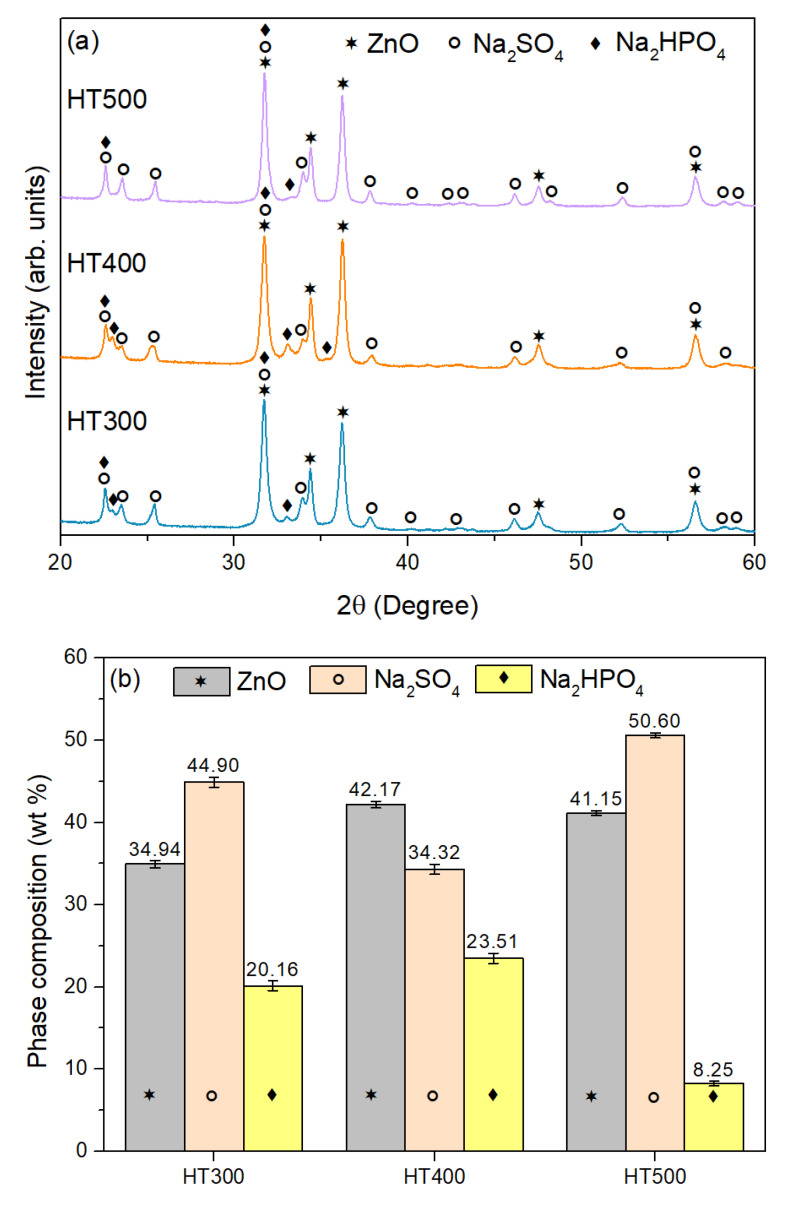

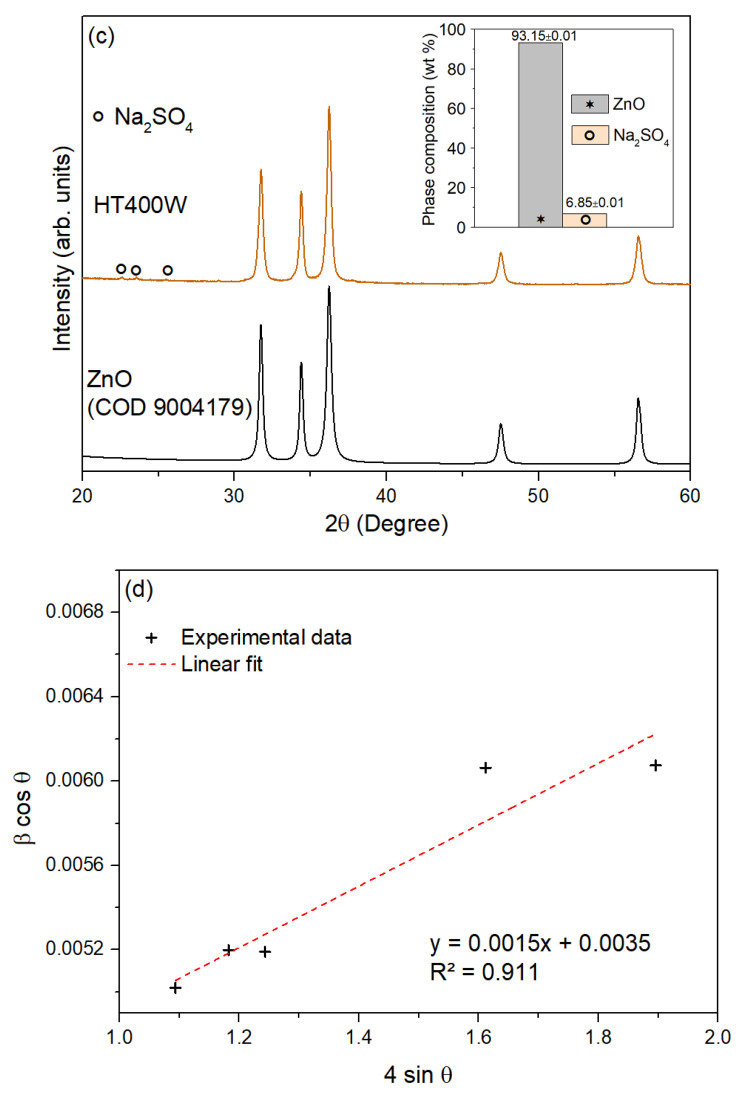
(**a**) XRD diffractogram of the sintered samples HT300, HT400, and HT500; (**b**) Phase composition of the same samples, expressed as weight percentage, determined from the XRD patterns; (**c**) XRD diffractogram and phase composition (inset) of the washed sample, HT400W; (**d**) Williamson–Hall plot based on XRD data for the HT400W sample.

XRD analysis of the sintered samples (HT300, HT400, HT500) and of the washed sample (HT400W), conducted to investigate their crystalline phases and structural evolution, is depicted in Figure 4. The XRD patterns of the sintered samples (HT300, HT400, HT500) shown in Figure 4a reveal three primary crystalline phases, ZnO, Na_2_SO_4_, and Na_2_HPO_4_, identified by their characteristic peaks and indexed utilizing reference standards. These multi-phase compositions likely arise from precursor reactions. For ZnO, the reference pattern (COD 9004179) [42] indicates a hexagonal wurtzite structure (P6_3_mc), including prominent peaks for planes (100) at ~31.8°, (002) at ~34.5°, (101) at ~36.3°, (102) at ~47.6°, and (110) at ~56.6°, marked with stars (★). Na_2_SO_4_, based on COD 1010522 [43], exhibits an orthorhombic structure (Pnma) in its stable thenardite phase, with prominent plane peaks (200) at ~23.5°, (002) at ~28.9°, (211) at ~33.2°, (112) at ~40.6°, and (220) at ~43.8°, denoted by circles (○). Na_2_HPO_4_, referenced using ICDD 04-010-0181 [44], features a monoclinic structure (P2_1_/n), with significant reflections for planes such as (011) at ~16.6°, (112) at ~35.6°, (020) at ~42.0°, and (130) at ~47.3°, with black diamonds (♦).

The sintered samples show variations in phases compositions with increasing heat treatment temperature. From Figure 4b, it can determined that, for the HT300 sample, ZnO constitutes ~34.94 wt%. In comparison, Na_2_SO_4_ and Na_2_HPO_4_ account for approximately 44.90 wt% and 20.16 wt%, respectively, with a goodness-of-fit (GoF) of 3.36, indicating a moderate fit quality due to phase overlap [45]. In the HT400 sample, the ZnO phase increases to ~42.17 wt%, Na_2_SO_4_ reduces to ~34.32 wt%, and Na_2_HPO_4_ rises slightly to ~23.51 wt%, with a GoF of 3.52. For the HT500 sample, ZnO phase drops to ~41.15 wt%, Na_2_SO_4_ rises significantly to ~50.60 wt%, and Na_2_HPO_4_ falls to ~8.25 wt%, with a GoF of 2.57, reflecting an enhancement on the fit quality due to better phase separation [46]. The phase composition in Figure 4b highlights a notable rise in Na_2_SO_4_ from the HT300 sample to the HT500 sample, suggesting phase stabilization at higher temperatures. At the same time, Na_2_HPO_4_ decreases due to dehydration or decomposition, resulting from its thermal instability above 400 °C [47]. ZnO remains stable across the assessed temperature range, peaking at 400 °C. Figure 4c presents the XRD pattern for the washed sample (HT400W), with its phase composition shown in the inset. This sample exhibits a significant reduction in Na_2_SO_4_ and Na_2_HPO_4_, with ZnO dominating at ~93.15 wt% and Na_2_SO_4_ at ~6.85 wt%. The peaks align with ZnO’s reference (COD 9004179), displaying sharp reflections at (100), (002), (101), etc. planes, and minimal Na_2_SO_4_ peaks (e.g., (200) ~23.5°, (002) ~28.9°). Na_2_HPO_4_ peaks are absent, implying their removal during washing. The GoF for the HT400W sample is 2.36, indicating excellent fit quality due to the simplified ZnO-dominant phase [45,46]. The washing process selectively eliminates Na_2_SO_4_ and Na_2_HPO_4_, thereby enriching the ZnO content, due to their soluble nature in water. These observations are consistent with studies on ZnO and sodium salts under thermal processing, where crystallinity and phase stability improve with increasing temperature, and washing removes soluble phases [42,43]. In addition, these results are well aligned with the thermal analyses findings previously discussed.

The average crystallite size and lattice strain in the samples were studied using Williamson–Hall analysis, assuming the Uniform Deformation Model, where the strain is considered uniform in all crystallographic directions. This model assumes an isotropic nature of the crystal, meaning that the material properties are independent of the direction along which they are measured [48].

The quantitative analysis was carried out using Equation (1):

(1) $$\beta \;\cos\theta =\frac{N\lambda }{D}+4\;\varepsilon \;\sin\theta$$

where *D* is the crystallite size, *β* is the full width at half maximum (FWHM) of the diffracted peaks, *λ* is the wavelength of the X-ray radiation, *θ* is the diffraction angle, and *N* is a numerical factor, commonly referred to as the crystallite shape factor. According to the literature, *N* = 0.94 is considered a good approximation for ZnO [48,49].

As shown in Figure 4d, the term (*β*cos*θ*) was plotted against (4sin*θ*) for the preferred orientation peaks. Consequently, the slope of the fitted line represents the strain, and the y-intercept can be used to estimate the crystallite size. From this analysis, the lattice strain and crystallite size were determined to be approximately 0.15% and 41.4 nm, respectively. The relatively low lattice strain suggests minimal internal defects or dislocations in the crystal structure, reflecting good crystallinity [49].

**Figure 5:**
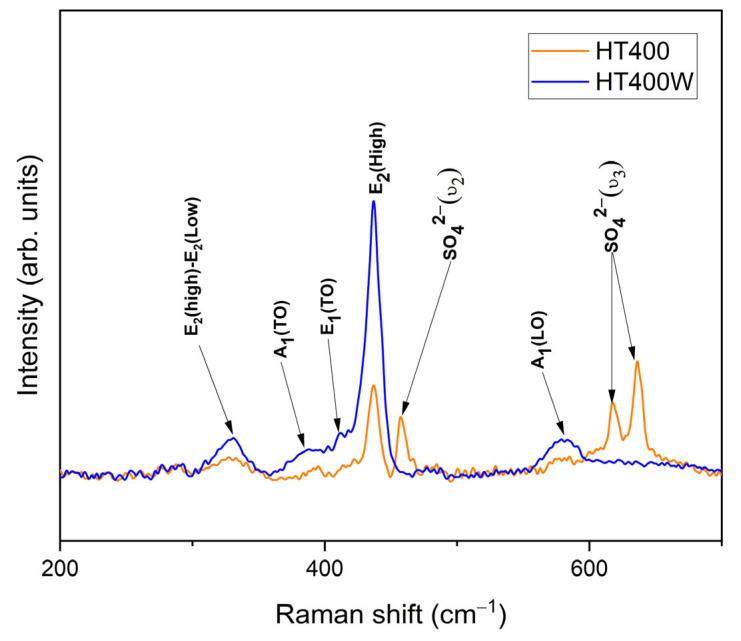
Raman spectra of the sintered sample before (HT400) and after washing (HT400W).

Raman spectroscopy was employed to investigate the vibrational modes and structural properties of the HT400 and HT400W samples, before and after washing, as presented in Figure 5. ZnO has a wurtzite structure with a C_6v_^4^ (P6_3_mc) space group, containing four atoms per unit cell, which results in Brillouin zone-center modes of Γ = 1A_1_ + 2B1 + 2E_2_ + 1E_1_ [28]. These include the polar A_1_ and E_1_ modes, and the two E_2_ modes (E_2_(low) and E_2_(high)). All these modes are Raman-active, while the B_1_ modes are silent. The A_1_ and E_1_ modes are polar, splitting into longitudinal optical (LO) and transverse optical (TO) phonons due to long-range electrostatic forces. In contrast, the E_2_ modes are nonpolar and associated with oxygen atom vibrations in the wurtzite lattice. The recognised Raman bands of ZnO are E_2_ (low) mode (100 cm^−1^), related to Zn sublattice vibrations, E_2_(high) mode (440 cm^−1^), associated with oxygen atom vibrations, A_1_(TO) mode (392 cm^−1^), E_1_(TO) mode (413 cm^−1^), and the A_1_/E_1_(LO) modes (580 cm^−1^), indicative of defects and oxygen vacancies [50].

For the sintered sample before washing, HT400, the Raman spectrum reveals a sharp peak centered at approximately 437 cm^−1^, corresponding to the nonpolar E_2_(high) mode, which provides a characteristic signature of crystalline ZnO [51]. However, the Raman spectrum reveals smaller peaks or shoulders, specifically at approximately 330 cm^−1^ (attributed to the E_2_(high)–E_2_(low) multiphonon process), 380 cm^−1^ (A_1_(TO) mode), 410 cm^−1^ (E_1_(TO) mode), and around 580 cm^−1^ (A_1_(LO) mode). Additionally, the peaks observed at 451 cm^−1^ (${SO}_{4}^{2-}\left(\vartheta_{2}\right)$) and within the 620–630 cm^−1^ region (${SO}_{4}^{2-}\left(\vartheta_{3}\right)$), both attributed to sulfate anion vibrational modes [52], suggest the presence of residual Na_2_SO_4_. This presence is likely due to byproducts formed during the green synthesis, possibly from sulfate-containing precursors like zinc sulfate, as corroborated by XRD data (Figure 4b).

Regarding the Raman spectrum of the washed sample, HT400W, depicted in Figure 5, allows us to observe significant improvements. Indeed, the E_2_ (high) peak at ~437 cm^−1^ remains the dominant feature but appears narrower and more intense, indicating reduced surface disorder due to the effective removal of byproducts during the washing process [53]. The remaining peaks, corresponding to the E_2_(high)–E_2_(low), A_1_(TO), E_1_(TO), and A_1_(LO) modes, demonstrate increased intensity, reflecting an enhancement in ZnO purity. Significantly, the peaks at 451, 620, and 630 cm^−1^ associated with Na_2_SO_4_ are completely removed, attesting to the successful removal of the secondary phases by washing. This observation is corroborated by the correspondent XRD analysis (Figure 4c), from which a predominant wurtzite ZnO phase with a phase composition of 93.15 wt% ZnO and only 6.85 wt% Na_2_SO_4_, was identified, indicating a near-pure ZnO structure with minimal residual sodium sulphate. The absence of Na_2_SO_4_ peaks in the Raman spectrum demonstrates the effectiveness of the washing step in enhancing sample purity, thereby improving structural integrity for applications that demand high-quality materials.

**Figure 6:**
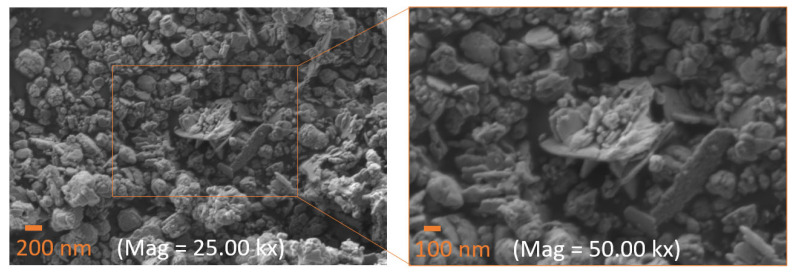
SEM micrographs of the HT400W sample at magnifications of 25.00 kx and 50.00 kx. Scale bars represent 200 nm and 100 nm, respectively.

Figure 6 presents SEM micrographs of the HT400W sample, allowing to assess its surface morphology at two magnifications. The left micrograph reveals a heterogeneous surface composed of irregularly shaped particles and aggregates, exhibiting a porous and rough texture, both characteristics of thermal processing and sintering. The right micrograph offers a detailed view with further magnification of the highlighted region (orange box), revealing a flake-like morphology. This structure suggests the presence of well-defined ZnO particles, likely formed during the heat treatment at 400 °C. The flakes consist of nanoscale features (ranging from 50 to 100 nm in width), with some areas displaying smoother surfaces and others retaining a rugged, porous structure, reflecting varying degrees of particle growth and aggregation.

Indeed, the cactus extract serves as a bio-template or growth-directing agent during the synthesis of ZnO, guiding nucleation and crystal growth pathways [54]. This effect is mainly attributed to the biomolecules in the extract, which function as natural capping and chelating agents. Organic compounds like polysaccharides and flavonoids interact with specific ZnO crystal faces, regulating their growth rates and leading to the formation of ZnO particles with diverse shapes and sizes. The viscous nature of the extract may also enhance particle adhesion, promoting the formation of asymmetrical clusters and contributing to the observed flake-like [55,56].

Using zinc sulfate as a precursor, Gopal and Kamila [57] achieved a similar ZnO flake or petal morphology after annealing at 400 °C, consistent with our findings. The observed morphology aligns with typical features of nanoparticles synthesized under identical conditions, where nanoparticles often form clusters due to their large surface area and high surface energy [56]. This agglomeration tendency is particularly pronounced in aqueous synthesis environments and green-synthesized NPs dynamics [34,58], which may apply to the preparation of the HT400W sample.

**Figure 7:**
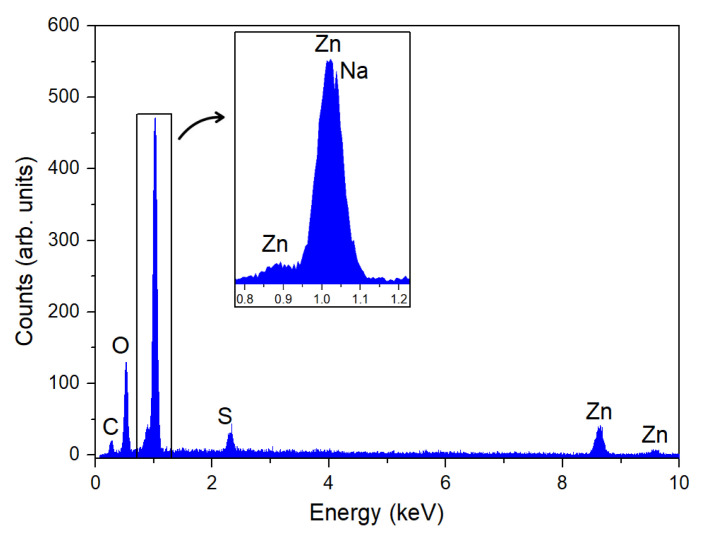
EDS spectrum of the HT400W sample.

Figure 7 shows the EDS spectrum of the HT400W sample. The spectrum reveals the presence of zinc (Zn), oxygen (O), sodium (Na), and sulfur (S), which aligns well with the phase composition determined by XRD analysis and further confirmed by Raman spectroscopy. The dominant Zn and O peaks correspond to the primary ZnO phase, confirming the successful synthesis of zinc oxide nanoparticles. The detection of sodium and sulfur peaks further supports the presence of the secondary phase Na_2_SO_4_, though in minor amounts, indicating the effectiveness of the washing procedure.

The carbon peak observed should be carefully interpreted, considering potential extrinsic contributions. It is important to distinguish whether carbon is a genuine constituent of the sample or an artifact arising from sample preparation.

Plant extracts, including those from cacti, contain various organic compounds. These organic molecules contain carbon, which can be incorporated into or remain on the surface of synthesized nanoparticles, especially in green synthesis routes. For instance, Farooqi et al. [59] assigned the carbon peak in the EDS spectrum to carbon-containing phytoconstituents adsorbed on the surface of silver nanoparticles synthesized using *Cicer arietinum* extract. Similarly, Bhavyasree et al. [60] associated the presence of the carbon EDS peak with biomolecules present in the aqueous leaf extract of *Adhatoda vasica* Nees used in the synthesis of CuO nanoparticles. In the same line, Patel et al. [61] attributed the high percentage of carbon detected in the EDS of iron oxide nanoparticles to the presence of biological molecules from the *Acacia jacquemontii* plant extract used during the synthesis process.

However, it is also important to consider that the sample was mounted using carbon tape—a common practice for securing specimens during EDS analysis. Carbon tape serves as a conductive adhesive but also introduces a carbon signal that may appear in the spectrum.

To study the dielectric and electrical properties of the HT400W sample, impedance spectroscopy measurements were performed at room temperature in the frequency range of 50 Hz to 10 MHz.

The complex impedance, Z*, can be represented as [62]:

(2) $$Z^{*}=Z^{\prime }-j\;Z^{\prime \prime }$$

where *Z*′ and *Z*″ are the real and imaginary parts of the complex impedance (corresponding to the resistance and reactance of the media), respectively.

**Figure 8:**
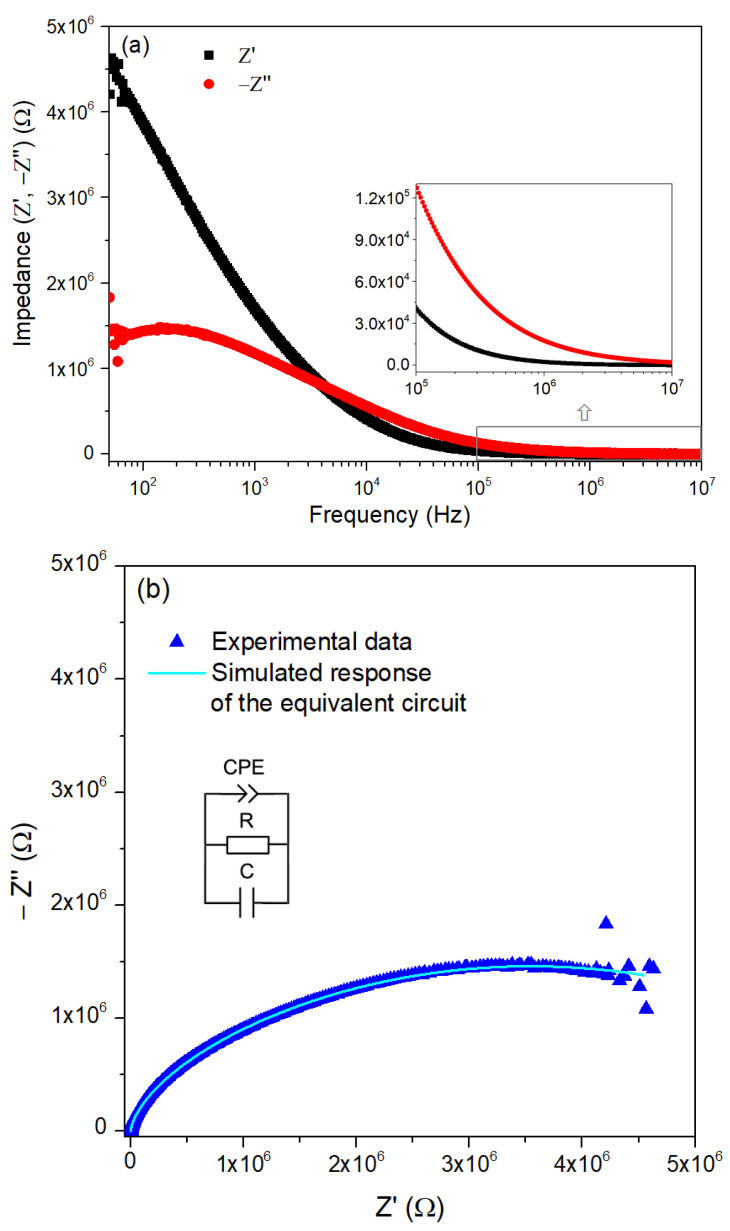
(**a**) Real, *Z*′, and imaginary, *Z*″, parts of the complex impedance, *Z*, and (**b**) Nyquist plot of the HT400W sample. The insert is the equivalent circuit.

Figure 8a shows the real and imaginary parts of the complex impedance for the measured frequency range. As the frequency increases, *Z*′ decreases monotonically, making the frequency dependence less pronounced in the high-frequency region. In turn, *Z*″ exhibits a clear peak, indicating the presence of a relaxation mechanism within the frequency range under investigation.

Figure 8b presents the corresponding Nyquist plot, where the blue triangles represent the experimental data, and the solid light blue line corresponds to the simulated response obtained for the equivalent circuit chosen as fitting model. The model, also depicted in Figure 8b, consists of a Constant Phase Element (CPE) in parallel with a resistor (R) and a capacitor (C), representing the non-ideal capacitive behavior observed in the impedance spectrum.

The observed semicircular shape is characteristic of a charge transfer resistance coupled with double-layer capacitance behavior [63]. The fitting was carried out using EIS Spectrum Analyzer Software, version 1.0. The excellent agreement between the experimental data and the simulated curve suggests that the selected equivalent circuit reliably represents the electrical behavior of the system.

Table 1 summarizes the estimated circuit parameter values obtained by fitting the experimental data using the mentioned simulation tool, considering that in the frequency domain, the impedance of a constant phase element is given by [64]:

(3) Z=Af−n(cosnπ2−jsinnπ2)

where A is the modulus of the impedance determined at the angular velocity of 1 rad/s, *f* is the frequency, and n is an exponent, equal to 1 for the case of a pure capacitor.

The frequency-dependent behavior of the dielectric permittivity, ε*, was also studied.

The dielectric permittivity is also a complex variable, which can be divided into the real part, ε′, and the imaginary part, ε″ [65]:

(4) $$\varepsilon^{*}=\varepsilon^{\prime }-j\;\varepsilon^{\prime \prime }$$

The dielectric constant, represented by the real component of permittivity, is associated with the material’s ability to store energy, while the imaginary part is related to the dielectric losses, which indicate the amount of dissipated energy [66].

The real and imaginary components of the complex permittivity can be obtained from the measured data using the following equations [67]:

(5) $$\varepsilon^{\prime }=\frac{-Z^{\prime \prime }d}{\omega \varepsilon_{0}A(Z^{\prime 2}+Z^{\prime \prime 2})}$$

(6) $$\varepsilon^{\prime \prime }=\frac{Z^{\prime }d}{\omega \varepsilon_{0}A(Z^{\prime 2}+Z^{\prime \prime 2})}$$

where *ε*_0_ represents the permittivity of free space (8.8542 × 10^−12^ F/m), *A* and *d* the area and thickness of the sample, respectively, and *ω *= 2π*f* the angular frequency.

The dielectric loss tangent can be obtained by:

(7) $$\tan\delta =\frac{\varepsilon^{\prime \prime }}{\varepsilon^{\prime }}$$

whereas, the AC conductivity, *σ_ac_*, can be extracted from the dielectric data using the equation [68]:

(8) $$\sigma_{AC=\varepsilon^{\prime \prime }\omega \varepsilon_{0}}$$

**Figure 9:**
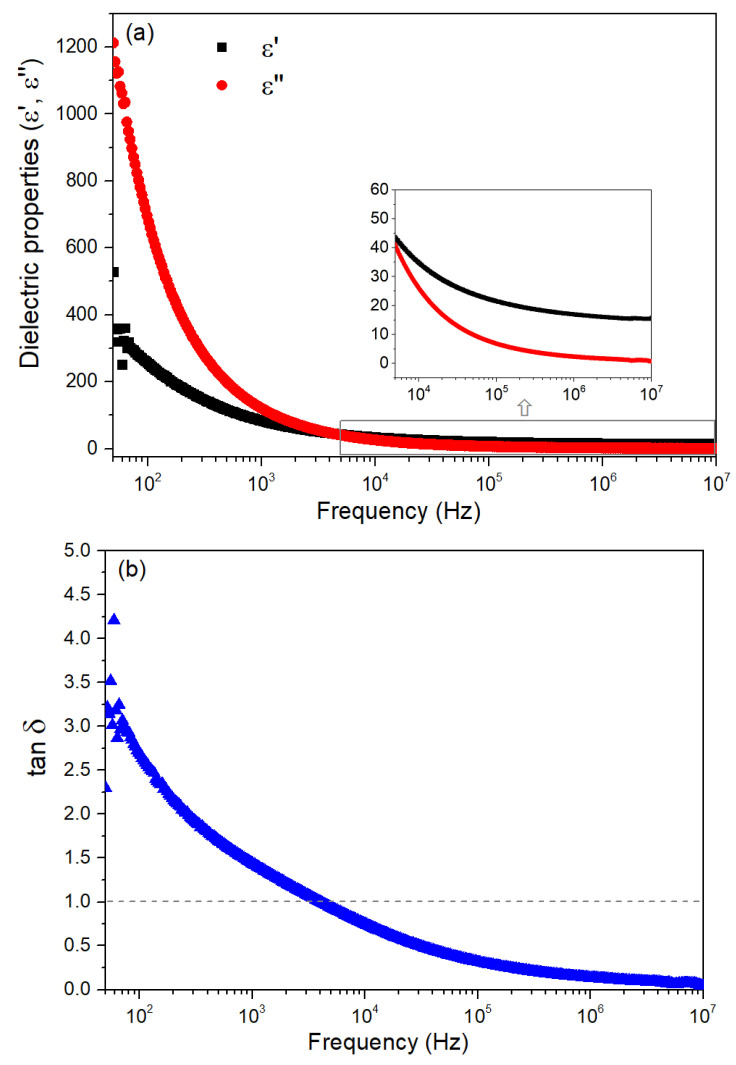
(**a**) Dielectric constant, ε′, dielectric loss, ε″ and (**b**) tan δ, of the HT400W sample.

Figure 9 presents the room temperature dielectric properties as a function of frequency for the HT400W sample, which presents a higher percentage of ZnO and improved crystallinity. Figure 9a illustrates the variation of the dielectric constant and dielectric losses over a frequency range from 50 to 10^7^ Hz, while Figure 9b shows the dielectric loss tangent over the same frequency range.

In Figure 9a, the dielectric constant and dielectric loss exhibit high values at low frequencies, which significantly decrease for the higher frequencies. This frequency-dependent behavior in ε′ aligns with the Maxwell–Wagner interfacial model, which is well-supported by Koop’s phenomenological theory for heterogeneous structures [69]. According to it, the HT400W sample likely consists of poorly resistive grains separated by highly resistive grain boundaries [70]. At low frequencies, charge carriers migrate into the grains and accumulate at the less conductive grain boundaries under an applied electric field, inducing significant interfacial polarization and resulting in a high dielectric constant [69]. This behavior is further explained by the collective contribution of various polarization mechanisms, including ionic, electronic, interfacial, and dipolar polarizations, which are prominent at lower frequencies [23]. However, as the frequency increases, the polarization diminishes because the dipoles cannot follow the rapidly oscillating electric field, leading to a substantial decline in ε′ and to a frequency-independent behavior at higher frequencies, a phenomenon often observed in ZnO materials due to rapid polarization processes [48,71]. Moreover, the inset of Figure 8a shows that within the frequency range up to 50 kHz, the dielectric loss is significantly lower than the dielectric constant. This indicates that the material has a lower energy dissipation compared to its energy storage capability.

In Figure 9b, the dielectric loss tangent value begins around four at 50 Hz, falls to 1 at 50 kHz, and then steadily decreases to about 0.14 at 1 MHz. This reduction in the tan δ with increasing frequency indicates lower energy losses at higher frequencies, a desirable trait for applications requiring minimal dielectric heating [72]. The high tan δ at low frequencies can be attributed to the contribution of space charge polarization, a consequence of the insulating interface formed at the grain boundaries [72], and the presence of defects or impurities, which are more active at lower frequencies [26,73]. As the frequency increases, these contributions diminish, resulting in reduced energy dissipation, which suggests its potential for practical use in energy storage technologies.

For easier assessment of the dielectric results, in Table 2 are summarized the dielectric properties of the obtained ZnO through green synthesis, which also simultaneously provides a comparison with data from other studies in the literature. The advantage of the current work lies in the balanced dielectric performance of the HT400W sample. The dieletric constant value, ε′, of 16.96 is higher than the ones reported by Lanje et al. [71], Zulfiqar et al. [73], and Taha et al. [72], and comparable to the one revealed by Dinesha et al. [74], reflecting effective polarization due to the green sol-gel method combined with the 400 °C calcination procedure, which enhances crystallinity and grain growth as observed through the XRD and SEM analyses. Notably, the tan δ of 0.14 is the lowest among all listed studies, indicating potentially improved energy efficiency and minimal dielectric loss. This low tan δ is a significant advantage compared to the higher tan δ values (0.21–0.8) observed in the remaining mentioned studies. While Saadi et al. [26] achieved a much higher ε′ value, their elevated ε″ and tan δ suggest greater energy dissipation, making the HT400W sample more suitable for applications requiring low loss, such as high-frequency electronic devices. The green sol-gel approach, combined with the optimized calcination and washing steps, thus offers a sustainable and effective synthesis route, highlighting the HT400W sample’s potential for advanced dielectric applications operating in the high-frequency range. 

## 4. Conclusions

This study successfully demonstrates the efficacy of the novel green synthesis route for ZnO nanoparticles utilizing *Hylocereus undatus* cladode extracts, where the key findings emphasize its potential as a sustainable alternative to traditional methods. TGA and DSC analyses further pinpointed thermal events, with a significant mass loss (24.62%) up to 100 °C due to water vaporization and ZnO formation at 272 °C, guiding us to the selection of calcination temperatures. The optimized synthesis, involving calcination at 400 °C followed by washing (HT400W), led to the formation of nanoparticles with a predominant wurtzite ZnO phase (93.15 wt%), alongside a minimal residual Na_2_SO_4_ impurity (6.85 wt%), effectively eliminating Na_2_HPO_4_. Raman spectroscopy revealed a sharp E_2_(high) peak at 437 cm^−1^ in the HT400W sample, indicating enhanced crystallinity and purity post-washing, with the removal of Na_2_SO_4_-related vibrational modes (451, 620–630 cm^−1^). SEM analysis disclosed a flake-like morphology with nanoscale features (50–100 nm), reflecting controlled particle formation facilitated by the phytochemicals in the cladode extract. The impedance data was fitted using an equivalent model circuit that consists of a Constant Phase Element (CPE) in parallel with a resistor (R) and a capacitor (C). The dielectric performance of the HT400W sample, exhibiting a dielectric constant (ε′) of 16.96 and a notably low loss tangent (tan δ) of 0.14 at 1 MHz, surpasses several literature benchmarks regarding energy efficiency. These findings demonstrate that this green approach can produce high-quality ZnO nanoparticles and deliver superior dielectric properties. Furthermore, the proposed eco-friendly approach for synthesizing ZnO nanoparticles with tailored physicochemical properties, positioned the obtained material as a promising candidate for various applications, ranging from biomedical to electronic uses.

## Figures and Tables

**Table 1:** Fitted results for the electrical parameters obtained from the equivalent circuit model used to describe the sample’s impedance behavior.

R (MΩ)	C (pF)	Q (ns)	n (a.u.)
7.57	8.34	6.97	0.447

**Table 2:** Dielectric properties of ZnO nanoparticles, determined at room temperature and 1 MHz.

Reference	Synthesis Method	Calcination Temperature	ε′	ε″	Tan δ
This work	Green sol-gel	400 °C	16.96	2.32	0.14
Lanje et al. [71]	Precipitation	100 °C	10.26	2.15	0.21
Dinesha et al. [74]	Combustion	400 °C	≈15	≈12	≈0.8
Saadi et al. [26]	Coprecipitation	500 °C	≈400	≈120	0.3
Zulfiqar et al. [73]	Chemical precipitation	600 °C	≈2.5	≈1	≈0.4
Taha et al. [72]	Sol-gel	550 °C	≈10	≈6	≈0.6

## Data Availability

The original contributions presented in this study are included in the article. Further inquiries can be directed to the corresponding author.
